# Perceptions of the benefits of the basic medical insurance system among the insured: a mixed methods research of a northern city in China

**DOI:** 10.3389/fpubh.2023.1043153

**Published:** 2023-04-17

**Authors:** Peng Wang, Shuyi Li, Zhizhen Wang, Mingli Jiao, Yuchao Zhang, Weiqi Huang, Ning Ning, Lijun Gao, Linghan Shan, Ye Li, Qunhong Wu

**Affiliations:** ^1^Department of Social Medicine, School of Health Management, Harbin Medical University, Harbin, China; ^2^School of Public Health, Harbin Medical University, Harbin, China; ^3^Research Center of Health Policy and Management, School of Health Management, Harbin Medical University, Harbin, China

**Keywords:** basic medical insurance system, insured, perceptions of benefits, policy literacy, mixed methods research

## Abstract

**Background:**

The perceptions of the benefits of the basic medical insurance system among the insured not only reflect the system's performance but also the public's basic medical insurance policy literacy, valuable information for countries that have entered the stage of deepening reform. This study aims to examine the factors that affect the perceptions of the benefits of the basic medical insurance system in China, diagnose the key problems, and propose corresponding measures for improvement.

**Methods:**

A mixed method design was used. Data for the quantitative study were obtained from a cross-sectional questionnaire survey (*n* = 1,045) of residents of Harbin who had enrolled for basic medical insurance system. A quota sampling method was further adopted. A multivariate logistic regression model was then employed to identify the factors influencing the perceptions of the benefits of the basic medical insurance system, followed by semi-structured interviews with 30 conveniently selected key informants. Interpretative phenomenological analysis was used to analyze the interview data.

**Results:**

Approximately 44% of insured persons reported low perceptions of benefits. The logistic regression model showed that low perceptions of the benefits of the basic medical insurance system was positively correlated with the experience of daily drug purchases (OR = 1.967), perceptions of recognition with basic medical insurance system (OR = 1.948), perceptions of the financial burden of participation costs (OR = 1.887), perceptions of the convenience of using basic medical insurance for medical treatment (OR = 1.770), perceptions of the financial burden of daily drug purchases costs (OR = 1.721), perceptions of the financial burden of hospitalization costs (OR = 1.570), and type of basic medical insurance system (OR = 1.456). The results of the qualitative analysis showed that the key problem areas of perceptions of the benefits of the basic medical insurance system were: (I) system design of basic medical insurance; (II) intuitive cognition of the insured; (III) rational cognition of the insured; and (IV) the system environment.

**Conclusions:**

Improving the perceptions of the benefits of the basic medical insurance system of the insured requires joint efforts in improving system design and implementation, exploring effective publicity methods of basic medical insurance system information, supporting public policy literacy, and promoting the health system environment.

## 1. Introduction

Medical insurance systems are often used to improve the accessibility and equity of health services and to protect their populations from medical poverty (1). Globally, many countries regard it as an important part of the social security system and promote universal coverage (2, 3). The insured are not only the target group of the system, but also the actual users and final evaluators. Their perceptions and evaluations of benefits are a comprehensive reflection of many factors, such as objective system design and implementation (4), subjective expectation (5), cognition and emotion as well as practical needs (6, 7). Making the public truly experience the benefits is an important goal of medical insurance reform. It is important and necessary to systematically examine the current situation, the influencing factors and key problems of the perceptions of benefits among the insured.

China has made significant progress in the construction and development of basic medical insurance systems (BMIS) in many aspects. Initially, the country provided economic security for more than 1.3 billion people through the establishment of three basic systems: namely, the urban employee basic medical insurance (UEBMI) for urban employees, the urban resident basic medical insurance (URBMI) for unemployed urban residents, and the new rural cooperative medical scheme (NCMS) for rural residents (8). After basically realizing universal coverage (>95% of the population), China integrated URBMI and NCMS into the urban and rural resident basic medical insurance (URRBMI) scheme (9) to improve the fund's risk sharing ability, management efficiency, and institutional fairness. Regarding service coverage, the country has implemented the dynamic adjustment policy of a drug catalog and has gradually optimized the types and quantity of reimbursable drugs (10). Moreover, to cooperate with the hierarchical medical system, the government has formulated the differentiated reimbursement policy of BMIS to reasonably guide patients to different medical institutions, which increases the access to and efficiency of medical services (11). Regarding health service cost coverage, the country has conducted a reform of the payment method of BMIS to control the increase in medical expenses (12). To improve the level of welfare, the Chinese government has increased financial subsidies and is expanding the proportion of reimbursement yearly (13). Additionally, the government has worked to enhance the support policies of BMIS at the national level, such as by establishing a legal system to ensure the safety of funds (14). Overall, the Chinese BMIS is working in a people-centered direction and is playing a vital role in improving the level of benefits for the insured.

However, existing evidences suggest that these efforts have not been effective in increasing the subjective perceptions and evaluations of the insured. Jing et al. (15) conducted a questionnaire survey with 3,231 insured people and found that 51.3% of them had medium or low satisfaction with BMIS. According to Liu et al. (16), the insured are generally dissatisfied with the quality of BMIS policies. Shan et al. (5) reached similar conclusions and found that the situation of BMIS is far from the expectations and preferences of the insured. Additionally, many scholars have confirmed this view from different angles. Tao et al. (17) showed that China's out of pocket expenditures (OOPE) decreased significantly, from 60.13% in 2000 to 35.91% in 2016, but were significantly higher than those of OECD member countries. The number of OOPE per capita continues to increase. Accordingly, the economic pressure of the insured is still serious (18). Shan et al. (5, 19) showed that many insured had low perceptions of the fairness and convenience of BMIS. The insured's perceptions of the key performance of BMIS reform can reflect the shortcomings of the reform and the problems that require key intervention in the future.

Regarding the insured's perceptions of the benefits of the basic medical insurance system (PBBMI), most scholars have explored the system design problems of the current BMIS via quantitative research methods, such as the poor protection effect of medical insurance on the seriously ill population (20), the insufficient protection on the fairness of different groups (21), the poor publicity effect of medical insurance information (22), and the poor portability of medical insurance (5). In most related qualitative studies, scholars have studied BMIS by interviewing system administrators or the insured. The administrators mainly focused on the management, financing, population, welfare design, structure, operating procedures, and interaction with health service providers (23, 24). The insured mainly focused on the coverage of BMIS and the fairness of services (25–27). Although previous research has made valuable efforts, there are still some deficiencies in the existing literature. First, most scholars have focused on a single dimension of BMIS and lack an evaluation of the perceptions of benefits of comprehensive medical insurance performance in multiple dimensions. Second, subjective cognitive factors of the insured are rarely discussed (28). Third, few studies have used mixed-methods research, which can overcome certain limitations of quantitative and qualitative research and allow researchers to fully capture the complexity of measurement and obtain reliable findings (29). Fourth, previous scholars often equate satisfaction with perceptions of benefits (5). However, BMIS satisfaction has evolved from commercial customer satisfaction (30), which is more biased toward evaluating BMIS as a private product similar to ordinary goods, and therefore hardly reflects the public benefits of BMIS. Based on the foundation and shortcomings of previous scholars, this study proposes the concept of PBBMI. Participants with different health status, health expectations, and socio-economic characteristics have overall perceptions of benefits of BMIS related to the needs of health services such as medical treatment, prevention, health care, and rehabilitation in the process of improving their own health. According to the key performance areas of BMIS, the composition of PBBMI should include the perceptions of financial burden, convenience, fairness, regulation, utility, awareness, and recognition.

This study aimed to answer the following question: Is the progress of China's BMIS in terms of coverage reflected in the PBBMI of the insured? By aiming to understand participants' overall perspective and by using a mixed-methods design, this study investigated which factors may affect the low PBBMI of the insured from a number of perspectives, such as varied socio-demographic status, health and disease status, recent medical insurance use and affairs-handling experience, and perceptions of key institutional performance indicators. Further, we aimed to determine the key issues in the system performance areas that have the greatest impact on the insured's PBBMI. This study's findings can provide more accurate intervention guidance for countries and regions where the medical insurance system is in development.

## 2. Materials and methods

### 2.1. Research design

This study was conducted in Harbin, China. Harbin is the capital of Heilongjiang Province, located in Northeast China, with a population of 9.885 million people. In 2021, the per capita GDP of Harbin was 53,517 yuan, ranking low among all provincial capitals in China. In general, the basic policies and regulations of China's BMIS are formulated by the national government, and local governments can make adjustments according to their own conditions without violating the basic principles. Policies at the national level generally regulate the basic structure of the national basic medical insurance, the reforms that must be implemented, and the minimum security standards. The capable local governments are allowed to make pilot explorations based on complying with the trend of national BMIS reform. However, the regions that made pilot reforms of the system in advance account for a small proportion in China. The arrangement and reform of the BMIS in Harbin, Heilongjiang Province is consistent with most regions in China and essentially mirrors national basic policies and regulations, which can reflect some common phenomena and problems to a certain extent.

A mixed research approach was used in this study. The quantitative study was aimed at identifying the factors that drove the insured's low PBBMI. The qualitative study however, explored the specific problem details of the key performance aspects of BMIS which were significant in the logistic regression results. Based on this, the key problem areas of low PBBMI are summarized. The results of the quantitative and qualitative studies were integrated to suggest improvements.

#### 2.1.1. Quantitative study design

**Figure 1:**
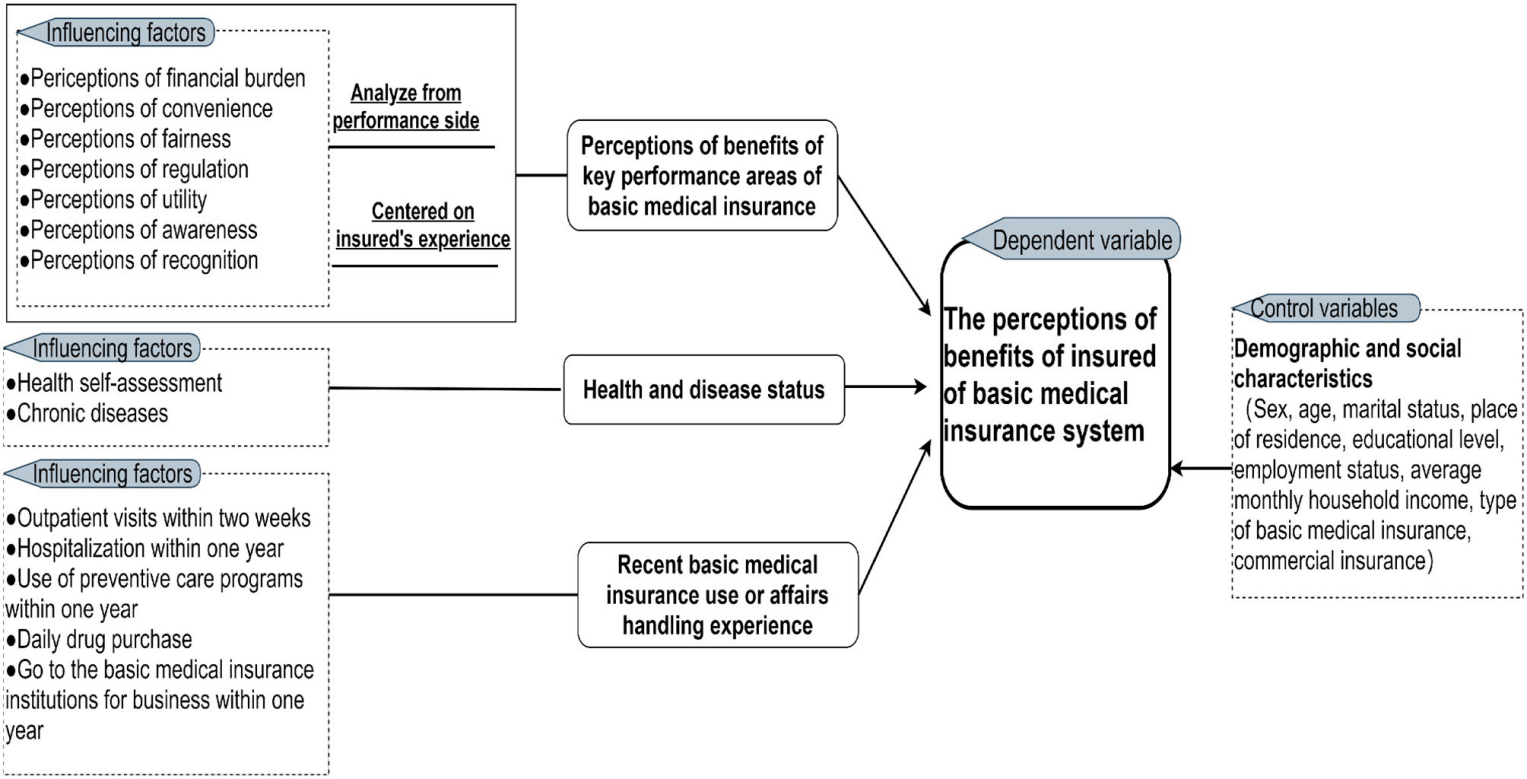
Analytical framework of the PBBMI of the insured.

An analytical framework (Figure 1) builds on several theories and research findings. User experience (UE) theory suggests that the products purchased and used should be evaluated based on user experience (31). Result-oriented performance evaluation theory emphasizes the evaluation of BMIS reform based on the policy performance (32). Additionally, many researchers have asserted that factors such as “financial burden, convenience, equity, regulation, utility, information awareness, and recognition” can significantly affect the insured's experience of the use of medical insurance (11, 16, 33–37). Jiang et al. (38) suggested that the “health status” of the insured is also a key factor in the perceptions of benefits. Moreover, Sadak et al. and Sanogo et al. (39, 40) have shown that the experience of the insured is fundamental to evaluating the perceptions of medical insurance benefits.

Based on this analytical framework, a 4-part questionnaire was developed. The first part gathered the demographic and socioeconomic characteristics of the insured. The second part evaluated the PBBMI of the key performance areas of the BMIS of the insured. The third part was aimed at evaluating the health status of the insured. The fourth part further investigated the BMIS use experience of the insured.

#### 2.1.2. Qualitative study design

Based on the seven key performance dimensions of BMIS in the quantitative questionnaire and relevant details in each dimension, an interview outline was developed. We aimed to collect information about major problems perceived by the insured in the key performance areas of BMIS, including the main problems felt about financial burdens (participation costs, outpatient costs, hospital costs, daily drug purchases costs), convenience (enrolment procedures, medical treatment, daily drug purchases, other business works), fairness (participation fees, health services, protection of treatment), regulation (insurance use behavior), utility (health services, primary treatment and two-way referral), awareness (accessibility of information), and recognition (the basic medical insurance system). In addition, we collected demographic and socioeconomic information of the respondents. During the interview, the interviewee was invited to make an overall evaluation of each of the seven dimensions, and was asked to give a detailed description of three problems of the BMIS that they perceived as the least beneficial (including, but not limited, to the above problems).

### 2.2. Sampling and data collection

#### 2.2.1. Quantitative data

A cross-sectional questionnaire survey was conducted during from December 2020 through February 2021. The survey respondents were selected using a quota sampling method to ensure representativeness. First, we determined balanced quotas for sex, age, education level, employment status, 2-week outpatient visits, hospitalization in the last year, and chronic disease incidence based on population estimates provided by the Heilongjiang Statistical Yearbook (41), China Health Statistical Yearbook (42), China National Health and Nutrition Big Data Report (43), and our past research experience. Three suitable communities in Harbin were then selected for the survey using a convenience sampling method. The sample size was estimated based on the need for logistic regression analysis; thus, it was 10 times more than the number of independent variables. The number of survey respondents was proportional to the size of the selected communities.

The questionnaires were completed in person under the guidance of professional staff who were specially trained before the survey began. As this survey was conducted during the outbreak of COVID-19, the effect that resulted in a decrease in the number of rural population migrating to urban, and the utilization of outpatient and inpatient services by the insured compared to previous periods. A total of 1,063 residents who enrolled for BMIS completed the questionnaire. We excluded incomplete questionnaires and those with logical errors, resulting in a final sample size of 1,045. To ensure the quality of the questionnaire, Cronbach's alpha was used to evaluate the confidence quality level of the data. The Cronbach's alpha of this study was 0.926 (>0.9) (44), indicating good reliability.

#### 2.2.2. Qualitative data

Interviews were conducted between February and April 2021. The inclusion criteria included insured persons or those whose family members had recently used basic medical insurance. Respondents were selected through a convenience sampling method. Owing to the severe development of COVID-19 at that time, we conducted and recorded telephone interviews based on a semi-structured interview outline after obtaining verbal informed consent from the interviewees; the interviews lasted ~30–60 min. The number of interviewees was determined by the saturation level of the interview content; 30 people were eventually interviewed. The recorded interviews were converted into text for further study.

### 2.3. Data analysis

#### 2.3.1. Quantitative data

We conducted a multiple logistic regression analysis to determine the influencing factors of the PBBMI of the insured.

##### 2.3.1.1. Dependent variable

The PBBMI of the insured was the dependent variable. Participants responded on a five-point Likert scale (1 = completely disagree, 2 = disagree, 3 = neither disagree nor agree, 4 = agree, 5 = completely agree) to score the following statement: “Overall, I think the current PBBMI is very high.” The score was divided into two categories that is, 0 = low PBBMI (including complete disagree, disagree and neither disagree nor agree) and 1 = high PBBMI (including agree and completely agree) for the purpose of logistic regression modeling.

##### 2.3.1.2. Independent variables

###### 2.3.1.2.1. PBBMI of the key performance areas of BMIS

Participants responded on a five-point Likert scale (1 = completely disagree, 2 = disagree, 3 = neither disagree nor agree, 4 = agree, 5 = completely agree) to score the specific problems in the key performance areas of BMIS (including the perceptions of financial burden, convenience, fairness, regulation, utility, awareness and recognition). Table 1 shows these specific problems. In the regression modeling, the scores were transformed into a dichotomous measurement and coded as: 0 = poor (including complete disagree, disagree and neither disagree nor agree) and 1 = good (including agree and completely agree).

**Table 1:** Characteristics of respondents and overall PBBMI (*n* = 1,045).

**Characteristic of respondent**	***n* (%)**	**High PBBMI (%)**	**Low PBBMI (%)**	***X* ^2^**	***P*-value**
**Demographic and socioeconomic characteristics**					
Sex				0.434	0.510
Male	513 (49.1)	291 (56.7)	222 (43.3)		
Female	532 (50.9)	291 (54.7)	241 (45.3)		
Age (years)				2.736	0.603
1 ≤ 34	83 (7.9)	43 (51.8)	40 (48.2)		
35–44	217 (20.8)	127 (58.5)	90 (41.5)		
45–54	260 (24.9)	138 (53.1)	122 (46.9)		
55–64	270 (25.8)	157 (58.1)	113 (41.9)		
≥65	215 (20.6)	117 (54.4)	98 (45.6)		
Marital status				1.259	0.262
Married	796 (76.2)	451 (56.7)	345 (43.3)		
Other	249 (23.8)	131 (52.6)	118 (47.4)		
Place of residence				0.887	0.346
Urban	966 (92.4)	542 (56.1)	424 (43.9)		
Rural	79 (7.6)	40 (50.6)	39 (49.4)		
Level of education				24.217	0.000
Junior high school or below	398 (38.1)	190 (47.7)	208 (52.3)		
Senior high school	473 (45.3)	271 (57.3)	202 (42.7)		
Bachelor's or above	174 (16.6)	121 (69.5)	53 (30.5)		
Employment status				6.820	0.009
Employed	622 (59.5)	367 (59.0)	255 (41.0)		
Other	423 (40.5)	215 (50.8)	208 (49.2)		
Average monthly household income^a^				45.853	0.000
1	242 (23.2)	106 (43.8)	136 (56.2)		
2	183 (17.5)	90 (49.2)	93 (50.8)		
3	227 (21.7)	118 (52.0)	109 (48.0)		
4	201 (19.2)	129 (64.2)	72 (35.8)		
5	192 (18.4)	139 (72.4)	53 (27.6)		
Type of BMIS				22.870	0.000
UEBMI	547 (52.3)	343 (62.7)	204 (37.3)		
URRBMI	498 (47.7)	239 (48.0)	259 (52.0)		
Commercial insurance				31.568	0.000
Not purchased	840 (80.4)	432 (51.4)	408 (48.6)		
Purchased	205 (19.6)	150 (73.2)	55 (26.8)		
**PBBMI of the key performance areas of BMIS**					
**Perceptions of financial burden**					
Participation costs				169.051	0.000
Poor	417 (39.9)	130 (31.2)	287 (68.8)		
Good	628 (60.1)	452 (72.0)	176 (28.0)		
Outpatient costs				131.444	0.000
Poor	544 (52.1)	211 (38.8)	333 (61.2)		
Good	501 (47.9)	371 (74.1)	130 (25.9)		
Hospital costs				122.171	0.000
Poor	531 (50.8)	207 (39.0)	324 (61.0)		
Good	514 (49.2)	375 (73.0)	139 (27.0)		
Daily drug purchases costs				144.206	0.000
Poor	564 (54.0)	218 (38.7)	346 (61.3)		
Good	481 (46.0)	364 (75.7)	117 (24.3)		
**Perceptions of convenience**					
Enrolment procedures				122.853	0.000
Poor	336 (32.2)	104 (31.0)	232 (69.0)		
Good	709 (67.8)	478 (67.4)	231 (32.6)		
Medical treatment				142.483	0.000
Poor	314 (30.0)	87 (27.2)	227 (72.3)		
Good	731 (70.0)	495 (67.7)	236 (32.3)		
Daily drug purchases				86.751	0.000
Poor	293 (28.0)	96 (32.8)	197 (67.2)		
Good	752 (72.0)	486 (64.6)	266 (35.4)		
Other business works				53.942	0.000
Poor	574 (54.9)	261 (45.5)	313 (54.5)		
Good	471 (45.1)	321 (68.2)	150 (31.8)		
**Perceptions of fairness**					
Participation fees				70.848	0.000
Poor	575 (55.0)	253 (44.0)	322 (56.0)		
Good	470 (45.0)	329 (70.0)	141 (30.0)		
Health services				58.750	0.000
Poor	622 (59.5)	286 (46.0)	336 (54.0)		
Good	423 (40.5)	296 (70.0)	127 (30.0)		
Protection of treatment				50.093	0.000
Poor	633 (60.6)	297 (46.9)	336 (53.1)		
Good	412 (39.4)	285 (69.2)	127 (30.8)		
**Perceptions of regulation**					
Insurance use behavior				4.557	0.033
Poor	759 (72.6)	438 (57.7)	321 (42.3)		
Good	286 (27.4)	144 (50.3)	142 (49.7)		
**Perceptions of utility**					
Health services				100.146	0.000
Poor	391 (37.4)	140 (35.8)	251 (64.2)		
Good	654 (62.6)	442 (67.6)	212 (32.4)		
Primary treatment and two-way referral				93.356	0.000
Poor	380 (36.4)	137 (36.1)	243 (63.9)		
Good	665 (63.6)	445 (66.9)	220 (33.1)		
**Perceptions of awareness**					
Accessibility of information				26.787	0.000
Poor	561 (53.7)	271 (48.3)	290 (51.7)		
Good	484 (46.3)	311 (64.3)	173 (35.7)		
Perceptions of recognition				164.545	0.000
**The basic medical insurance system**					
Poor	332 (31.8)	89 (26.8)	243 (73.2)		
Good	713 (68.2)	493 (69.1)	220 (30.9)		
**Health and disease status**					
Health self-assessment				4.640	0.031
Poor	576 (55.1)	338 (58.7)	238 (41.3)		
Good	469 (44.9)	244 (52.0)	225 (48.0)		
Chronic diseases				2.347	0.126
No chronic diseases	859 (82.2)	469 (54.6)	390 (45.4)		
With chronic diseases	186 (17.8)	113 (60.8)	73 (39.2)		
**Recent basic medical insurance use or affairs handling experience**					
Outpatient visits within 2 weeks				23.462	0.000
No experience	990 (94.7)	534 (53.9)	456 (46.1)		
Have experience	55 (5.3)	48 (87.3)	7 (12.7)		
Hospitalization within 1 year				7.908	0.005
No experience	988 (94.5)	540 (54.7)	448 (45.3)		
Have experience	57 (5.5)	42 (73.7)	15 (26.3)		
Use of preventive care programs within 1 year				17.324	0.000
No experience	944 (90.3)	506 (53.6)	438 (46.4)		
Have experience	101 (9.7)	76 (75.2)	25 (24.8)		
Daily drug purchase				34.419	0.000
No experience	189 (18.1)	69 (36.5)	120 (63.5)		
Have experience	856 (81.9)	513 (59.5)	343 (40.1)		
Go to the basic medical insurance institutions for business within 1 year				0.800	0.371
No experience	957 (91.6)	529 (55.3)	428 (44.7)		
Have experience	88 (8.4)	53 (60.2)	35 (39.8)		

a Quintile 1 is the poorest and quintile 5 is the wealthiest.

BMIS, basic medical insurance system.

###### 2.3.1.2.2. Health and disease status

Survey respondents were asked to answer the following question: “Out of 100, how would you rate your current health status?” and “Do you have a chronic disease?” In the regression modeling, the median method was used to classify the health self-ratings into 2 categories: 0 = poor ( ≤ 85) and 1 = good (>85). The chronic disease status was divided into 2 categories: 0 = no chronic disease and 1 = with chronic disease.

###### 2.3.1.2.3. Recent basic medical insurance use or affairs handling experience

Survey respondents were asked to answer the following question: “Do you have following experience: outpatient visits within two weeks, hospitalization within one year, preventive care within one year, daily drug purchases and go to the basic medical insurance institutions for business within one year?” In the regression modeling, participants' responses were divided into 2 categories: 0 = no experience and 1 = have experience.

###### 2.3.1.2.4. Control variables

In the statistical analysis, we controlled the confounding effect of the demographic and socioeconomic characteristics (age, gender, marital status, education, etc.) of the survey respondents.

##### 2.3.1.2. Statistical analysis

Data were further analyzed using SPSS 25.0. We initially determined the relationship between the PBBMI and each independent variable using the Chi-square test. We then constructed two regression models, one including all independent variables (Hosmer-Lemeshow test, X^2^ = 7.535, *p* = 0.480) and the other including only those variables that were statistically significant (*p* < 0.05) in the Chi-square test (Hosmer-Lemeshow test, X^2^ = 6.324, *p* = 0.611). The second model showed a better fit compared to the first one and slightly different odds ratios (ORs) compared with the first one. Accordingly, we only show the results of the second model.

#### 2.3.2. Qualitative data

Interpretative phenomenological analysis (IPA) was performed with the Interview materials (45). In the first step, the six researchers were divided into three groups of two persons each; each group conducted interviews with different interviewees. In the second step, the interviewers converted the recordings into text and read them several times to ensure the consistency between the text and the recordings after each interview, and further held regular group meetings to discuss the content of each group's interview. In the case the content of the interview was found to be detached from the research topic or saturated with content, the interview would be adjusted or stopped. In the third step, to ensure consistency in the analysis of textual information, we selected only two researchers to tag and code textual information related to the low PBBMI of the insured. In addition, in this step, we combined the results of quantitative analysis to eliminate irrelevant content in the qualitative data, so as to ensure the consistency of the interpretation of the qualitative results to the quantitative results. In the fourth step, the two researchers repeatedly thought about the connections between the tags and codes of the filtered text content, gathered the connected tags and codes together, summarized and refined them into primary themes, and then held regular group meetings to discuss these themes until a consensus was reached. In the fifth step, the primary themes were summarized and refined to form more representative secondary and tertiary themes.

## 3. Results

### 3.1. Quantitative research phase

#### 3.1.1. Characteristics of respondents and the PBBMI of the insured

The number of female respondents was approximately equal to that of male (50.9%); the majority of respondents were 64 years old or younger (79.4%); married (76.2%); lived in cities (92.4%); had no bachelor degree or higher (83.4%); and were employed (59.5%). More than half of the respondents were UEBMI (52.3%) and a small proportion of respondents had commercial insurance (19.6%).

Overall, ~44% of the insured had low PBBMI. The Chi-square tests showed that the PBBMI was associated with the demographic and socioeconomic characteristics of respondents, the perceptions of key performance areas of BMIS, health status and the use experience of recent basic medical insurance. The respondents who had a lower level of education, were unemployed, and whose average monthly household income is the poorest had lower PBBMI. Those who enrolled for URRMBI and have no commercial insurance are more likely to have a low PBBMI. Apart from the perceptions of regulation, the respondents whose perceptions of the key performance areas of BMIS are poor are more likely to have low PBBMI. Moreover, the respondents whose health self-assessment is good also exhibited lower PBBMI. Those who had no recent use experience of basic medical insurance exhibited lower PBMMI (Table 1).

#### 3.1.2. Logistic regression model

After controlling for confounding factors, the logistic regression model identified seven influencing factors (*p* < 0.05) of PBBMI: the experience with daily drug purchases (OR = 1.967), the perceptions of the recognition of BMIS (OR = 1.948), the perceptions of the financial burden of participation costs (OR = 1.887), the perceptions of the convenience of medical treatment (OR = 1.770), the perceptions of the financial burden of daily drug purchases costs (OR = 1.721), the perceptions of the financial burden of hospitalization costs (OR = 1.570) and the type of BMIS (OR = 1.456). Table 2 presents the details.

**Table 2:** Logistic regression analysis on the PBBMI.

**Variable**	**Walds**	***P*-value**	**OR**	**95% CI**	
**Demographic and socioeconomic characteristics**					
Level of education	4.793	0.091			
Senior high school	0.601	0.438	1.148	0.809	1.629
Bachelor's or above	4.736	0.030	1.767	1.058	2.950
Junior high school or below (reference)					
**Employment status**					
Employed	0.031	0.860	0.970	0.692	1.360
Other (reference)					
**Average monthly household income** ^**a**^	7.466	0.113			
2	0.045	0.833	1.053	0.650	1.706
3	0.109	0.741	1.082	0.677	1.729
4	2.840	0.092	1.539	0.932	2.543
5	5.209	0.022	1.844	1.090	3.117
1 (reference)					
**BMIS**					
UEBMI	5.358	0.021	1.456	1.059	2.001
URRBMI (reference)					
**Commercial insurance**					
Purchased	1.639	0.200	1.322	0.862	2.026
Not purchased (reference)					
**PBBMI of the key performance areas of BMIS**					
**Perceptions of financial burden**					
**Participation costs**					
Good	12.717	0.000	1.887	1.331	2.675
Poor (reference)					
**Outpatient costs**					
Good	1.757	0.185	1.292	0.885	1.885
Poor (reference)					
**Hospital costs**					
Good	6.147	0.013	1.570	1.099	2.242
Poor (reference)					
**Daily drug purchases costs**					
Good	8.891	0.003	1.721	1.204	2.458
Poor (reference)					
**Perceptions of convenience**					
**Enrolment procedures**					
Good	0.032	0.859	0.964	0.642	1.447
Poor (reference)					
**Medical treatment**					
Good	7.431	0.006	1.770	1.174	2.667
Poor (reference)					
**Daily drug purchases**					
Good	2.027	0.155	1.326	0.899	1.957
Poor (reference)					
**Other business works**					
Good	0.415	0.519	1.124	0.788	1.604
Poor (reference)					
**Perceptions of fairness**					
**Participation fees**					
Good	0.251	0.617	1.112	0.734	1.684
Poor (reference)					
**Health services utilization**					
Good	0.256	0.613	1.114	0.733	1.692
Poor (reference)					
**Protection level**					
Good	1.221	0.269	0.783	0.508	1.208
Poor (reference)					
**Perceptions of regulation**					
**Basic medical insurance use behavior**					
Good	0.273	0.602	0.910	0.639	1.296
Poor (reference)					
**Perceptions of utility**					
**Health services utilization**					
Good	0.856	0.355	1.192	0.822	1.729
Poor (reference)					
**Primary treatment and two-way referral**					
Good	3.653	0.056	1.413	0.991	2.013
Poor (reference)					
**Perceptions of awareness**					
**Accessibility of information**					
Good	0.247	0.619	0.910	0.628	1.319
Poor (reference)					
**Perceptions of recognition**					
**The basic medical insurance system**					
Good	10.743	0.001	1.948	1.307	2.903
Poor (reference)					
**Health status**					
Health self-assessment					
Good	3.037	0.081	0.727	0.508	1.041
Poor (reference)					
**Recent basic medical insurance use experience**					
**Outpatient visits within 2 weeks**					
Have experience	1.924	0.165	2.037	0.745	5.567
No experience (reference)					
**Hospitalization within 1 year**					
Have experience	0.313	0.576	1.265	0.555	2.886
No experience (reference)					
**Use of preventive care programs within 1 year**					
Have experience	1.659	0.198	1.471	0.818	2.644
No experience (reference)					
**Daily drug purchase**					
Have experience	10.178	0.001	1.967	1.298	2.980
No experience (reference)					
**Constants**	55.680	0.000	0.062		

a Quintile 1 is the poorest and quintile 5 is the wealthiest.

BMIS, basic medical insurance system.

### 3.2. Qualitative research phase

Qualitative interviews mainly diagnosed specific problem details of the key performance areas of BMIS, which was significant in the quantitative research. Table 3 shows the main themes that emerged from the analysis. To promote understanding, we have included quotations in Supplementary material 1.

**Table 3:** Main themes.

**Tertiary theme**	**Secondary theme**	**Primary theme**
Basic medical insurance system design	Inadequate coverage of services and guarantees	High drug price
		Inadequate reimbursement of drugs and medical consumables
	Insufficient attention to the needs of low-income people	The burden of participation costs
		The burden of advancing payment of hospitalization costs
	Lack of regulatory measures and support	Lack of supervision and intervention in the risk of irregularities by medical institutions and doctors
		Lack of guarantee of drug supply
Intuitive cognitive bias	Non-status quo reference point selection	Expect the cost of participation will remain the same and hospitalization will be fully reimbursed
	Availability heuristics	Reliance on relatives and friends
	Representativeness heuristics	Doctors' omniscient Inference
Rational cognitive bias	Lack of information	Incomplete access to effective information about limited payment policy
		Information update lag about medical treatment in non-residential places
	Misinterpretation of information	Media misleading
		The quality signals released by the basic medical insurance catalog adjustment are ineffectively received
	Misjudgment of role and function	Participating in indifference
		Basic medical insurance card function misjudgment
System environmental	Health system performance	Poor attitude of doctors
		The hierarchical medical system is ineffective
	Medical Information Development	Older adults have difficulty adapting to health care information technology

## 4. Discussion

This exploratory study showed that 44% of the insured exhibited low PBBMI. The quantitative research showed that the PBBMI was associated with the demographic and socioeconomic characteristics of respondents (type of MBIS), recent basic medical insurance use experience (daily drug purchases) and the perceptions of the key performance areas of BMIS (recognition of BMIS, financial burden of participation costs, convenience of medical treatment, financial burden of daily drug purchases costs, financial burden of hospitalization costs). Qualitative research identifies four main areas of the problems about the key performance of BMIS that trigger low PBBMI: (I) the system design of basic medical insurance; (II) the intuitive cognition of the insured; (III) the rational cognition of the insured; and (IV) the system environment.

### 4.1. Characteristics of the insured with low PBBMI

This study shows that compared with the UEBMI, the insured who enrolled in the URRBMI had lower PBBMI (OR = 1.456). Although the system integration improved the welfare level of the URRBMI (38), the difference between residents' and employees' contributory capacity means that URRMI and UEBMI in institutional treatment differ significantly, reducing the PBBMI of the insured who enrolled for the URRMI.

Additionally, the results of the chi square test showed that the insured who lacked recent basic medical insurance use experience had lower PBBMI. The public's perceptions of reform and change are lagging, especially in areas where they do not enter frequently (22). The insured who had not used medical care recently were more likely to be affected by the collective memory of the once extremely insufficient medical security in Heilongjiang (46, 47). Among the specific types of usage experiences, daily drug purchase experience was statistically significant in the dimension of recent basic medical insurance use experience (OR = 1.967 compared with no daily drug purchase experience). This was likely because compared with other basic medical insurance use experiences, the insured have higher expectations for inexpensive daily drug purchases. However, the prices in drugstores are now generally higher than in hospitals. This gap between expectation and reality can easily reduce individual's PBBMI.

### 4.2. Barriers to the PBBMI based on the key performance areas of BMIS

#### 4.2.1. System design of basic medical insurance

Scientific system design is not only the premise of the smooth operation and effectiveness of the system, but also a significant influencing factor of PBBMI (48). We found that the problems about key performance areas to which the insured responded were primarily related to the design of the BMIS, including inadequate coverage of guarantee, insufficient attention to the needs of low-income people, and a lack of regulatory measures and support.

##### 4.2.1.1. Inadequate coverage of services and guarantees

The coverage of services and guarantees has been the focus of reform. However, the price of drugs and the lack of reimbursable coverage of drugs and consumables are still issues that have triggered many complaints from the insured.

Of all the respondents, 54% thought that the price of medical drugs was high, which is consistent with the situation of China's drug service market (49). At the national level, expenditure on drugs has increased gradually (50). For example, in 2018, China's total drug expenditure was 1,914.89 billion yuan, accounting for 32.39% of the total health expenditure, which is far higher than the average of 17% in OECD countries (51). In addition, the results of a survey on drug prices in 31 Chinese provinces showed that drug prices in Heilongjiang province, the area we investigated, were generally higher than the national average (52). He et al. and Zeng et al. believe that this is related to the high price and quantity of drugs used (53, 54). China launched a volume procurement policy at the national level in 2018 to reduce drug prices in each province. The average price of 25 and 87 drug varieties selected in the next 2 years decreased by 59 and 53%, respectively (10, 55), which improved the above situation. However, the volume purchase policy at the national level is new, and the number of selected drugs is significantly less than that of drugs in the basic medical insurance catalog. Moreover, the policy does not fully cover retail pharmacies, and prices in pharmacies are generally higher than in hospitals. Therefore, drug prices do not meet the insured's expectations for immediate, low-cost purchases of various drugs, thus significantly affecting their PBBMI.

The interview results showed that some insured complained about the inadequate coverage of the basic medical insurance drug catalog and high-value medical consumables. According to existing research, insufficient coverage was mainly related to a few specific problems. First, China's health technology assessment is weak, which hinders the access of Medicare drugs and consumables (56, 57). Second, the dynamic adjustment mechanism of the basic medical insurance catalog is imperfect (58), and many safe and effective drugs or consumables have not been included in the reimbursable range. Harbin city implements the national medical insurance catalog standard, and the insufficient coverage has increased the medical economic burden and reduced the PBBMI of the insured. Therefore, these problems requires more attention in the future.

##### 4.2.1.2. Insufficient attention to low-income people

This study shows the BMIS does not sufficiently account for the low-income insured, which is reflected in the burden of insurance participation and advancing payment of hospitalization costs.

During the interviews, we found that the low-income insured expressed low PBBMI due to participation costs, which is mainly reflected in the absolute burden of the insured who enrolled for the URRBMI on the payment of participation cost, and the relative burden of the insured who enrolled for the two types of BMIS on the continuous rise of premiums. This finding is consistent with the results of another related study (16). Compared with the middle and high-income groups, the the low-income insured have faced great objective pressure in making daily payments for food, housing, education, and transportation. To reduce the insured's payment burden, the state has provided a large amount of financial subsidies to URRBMI. For example, in 2021, the financing standard of URRBMI in Harbin was 860 yuan, of which the state financial subsidy was 550 yuan per person. Individuals only needed to pay 310 yuan per year (59). However, the scarcity of disposable funds causes great economic pressure every time expenditure for the low-income insured is increased (38). Therefore, the government needs to pay high attention to the absolute burden of this part of the population in policy-making. Additionally, the financing standard of the participation costs of UEBMI and URRBMI shows an upward yearly trend (13, 16). Some interviewees with unstable and low income said that compared with previous years, the current higher amount of participation costs made it difficult to accept, and they experienced economic pressure. We further found that when determining the premium increase, these participants do not comprehensively consider some realistic conditions, such as the simultaneous growth of national finance and residents' disposable income. The data showed that the per capita disposable income of Harbin increased by about 34% from 2015 to 2020 (60). However, the interviewees were unaware of this increase and compared the current premium with the premium from previous years, resulting in a perception of economic burden. To address this way of thinking among the insured, the government may need to share medical insurance information publicity and increase education related to BMIS.

At present, Harbin, like most regions of China, implements the advance payment systems of hospitalization costs, that is, individuals are required to pay a certain amount of medical costs in advance before hospitalization, and then settle the actual amount incurred after the treatment. This study showed that the hospitalization advance payment system increased the hospitalization economic pressure of the low-income insured, consequently affecting their PBBMI. It is difficult for low-income people to suddenly pay a large hospital advance payment. In some cases, they are forced to reduce or abandon medical service (61). Moreover, the design of the advance payment system also increases the hidden opportunity cost (62).

##### 4.2.1.2.1 Lack of regulatory measures and support

Providing corresponding regulatory measures and support is an important part of the BMIS. This study shows that the insured think that there are still deficiencies in the supervision of medical institutions and doctors and the supply support of basic medical insurance drugs.

When the interviewees were asked about the problems affecting their perceptions of convenience of medical treatment, most of the insured mentioned the rules of limiting the duration of hospitalization, which also drove their low PBBMI. In Harbin, medical institutions set the standard for the average duration of hospitalization according to national regulations to control unreasonable basic medical insurance expenses and improve the utilization rate of medical resources (63). However, to obtain higher basic medical insurance compensation, some hospitals and doctors tackle the national regulations by decomposing hospitalization, ostensibly meeting the national standard for the duration of hospitalization (64). Patients need to bear the time and health cost of repeated discharge and hospitalization, resulting in poor medical experience and low PBBMI. Some scholars believe that these unreasonable medical behaviors are related to the lack of the BMIS regulatory system and regulatory tools (65). Although Harbin has followed national policies regarding supervision measures such as legislation, multi department coordination, social integrity system construction, and information construction in recent years, these methods are still in the preliminary exploration stage and need to be gradually improved.

Some insured indicated that they had to purchase drugs at retail pharmacies due to the shortage of hospital drugs, which drove their low PBBMI. This is consistent with other research results (66, 67). The shortage of basic medical insurance drugs in China is a complex problem with multiple factors. It is affected not only by drug procurement policies, but also by drug distribution and doctors' use habits. In the drug production chain, China's centralized drug procurement policy adopts the strategy of “the lowest price wins.” However, China has less control over the price of raw materials used to produce drugs and requires manufacturers to guarantee the quality of drugs. Accordingly, manufacturers face the risk of production costs outweighing profits and are consequently less motivated (68, 69), even abandoning bids or refusing to supply halfway through fulfilling an order. In the drug distribution chain, China implements the principle of exclusive distribution (67). If the distribution enterprise experiences transportation failure or delay, there will be a direct effect on the purchase and utilization of drugs. Regarding drug use, the ability of Chinese pharmacists to participate in the use of drugs by clinicians is limited, resulting in the lack of correction of some clinicians' irrational medication (70). These doctors are probably reluctant to use low-cost drugs, making their clinical dosage small, and causing enterprises to be reluctant to produce. Currently, many hospitals in Harbin suffer from a shortage of drugs, especially low-cost drugs (71). One of the priorities of China's medical reform is ensuring the supply of drugs. China needs to reinforce the guarantee and early warning of drugs, and improve the availability of drugs for participants.

#### 4.2.2. Intuitive cognitive bias

Intuitive cognition is generally considered a free, uncontrolled way of thinking. It can aid in rapid decision-making and saving cognitive resources, but it is affected by people's knowledge reserve, past experiences, and environment, and may therefore produce cognitive bias (72, 73). This study shows that when making actual judgments regarding BMIS, the insured usually lack accurate deductive reasoning, and are more affected by three main aspects: non-status quo reference dependence, accessibility heuristics, and representativeness heuristic. This results in the deviation between their overall experience of BMIS and their actual intuitive perceptions, which further affects their PBBMI.

##### 4.2.2.1. Non-status quo reference dependence—Expect the cost of participation will remain the same and hospitalization will be fully reimbursed

The concept of reference point originates from prospect theory proposed by Kahneman and Tversky, which suggests that people have comparative preferences in the process of decision making, potentially encoding the deviation direction and degree between the actual profit and loss of the decision-making result and the psychological central base point (reference point) (74), and make subsequent judgment accordingly. Generally, the reference point includes the current reference point with the actual situation as the reference and the non-current reference point with no objective situation as the reference, such as the individual's expectation or goal (75). During interviews, we found that the insured often take the expectation of non-rising participation costs and complete reimbursement of hospitalization costs as the non-current reference point. However, owing to the contradiction between people's growing medical and health care needs and limited BMIS resources, their actual experience is far from their actual expectations. From the perspective of reference, this unsatisfactory comparison result imperceptibly causes the participants to have cognitive bias and misjudge the BMIS, making the insured believe that they have incurred losses and have low perceptions of the recognition and benefits of the public welfare and utility of BMIS (76, 77).

##### 4.2.2.2. Availability heuristic—Relatives and friends dependence

The availability heuristic indicates that people tend to rely on information that is easily obtained and can be easily extracted from memory to make estimations and judgments. This method can make the information more accessible, but there is risk of biased judgments due to credulous misinformation (78). Specifically, we found that the perceptions and judgments of the insured regarding BMIS mostly come from information transmitted by relatives and friends, because this information is vivid and easy to understand and retrieve. However, these people typically do not know all the information related to BMIS, nor is the information they know necessarily true. Additionally, individualized BMIS usage plans are not the same for everyone. This information passed from friends and relatives can affect people's actual experiences and further low PBBMI.

##### 4.2.2.3. Representativeness heuristics—Doctors' omniscient inference

Representativeness heuristics indicates when people judge an event, they often choose representative cases and make inferences from them (78). We found that the insured have misperceptions about doctors' knowledge of BMIS because doctors generally have extensive medical-related knowledge and higher education (79). Additionally, doctors have close working knowledge of BMIS, so the insured tend to make conclusions based on their perception that doctors have excellent clinical knowledge and knowledge of BMIS policies. However, BMIS restricts and control doctors' diagnosis and treatment behavior. In recent years, the hospital payment method reforms of China's BMIS have made physicians do not stand the same position as BMIS many times (35). Additionally, doctors do not necessarily know BMIS details that patients want to know. Enrollees are often affected by the limited information provided by physicians, exaggerating the deficiency of BMIS.

#### 4.2.3. Rational cognitive bias

Compared with intuition, rationality can help people judge things more thoughtfully. However, in a complex social environment, it is impossible for a person to obtain all the information and knowledge relevant to their judgment. Moreover, the human brain has limitations in its ability to comprehend, calculate and analyze things, resulting in people's rational judgments being prone to errors (80). Our research shows that the main reason of BMIS rational judgment bias among the insured is the lack of information, misinterpretation of information and the misjudgment of roles and functions, resulting in low PBBMI.

##### 4.2.3.1. Lack of information

This study shows that incomplete access to information and information update lag could result in the bias of the rational cognition of the insured, which affects PBBMI. For example, during the interview, we found that insured's information about the restricted payment policy is inadequate, resulting in their misconception that the reimbursement rules for special drugs are the same as those for ordinary drugs. Some insured's understanding of hospitalization reimbursement policy of off-site medical treatment is still in the stage that the hospitalization expenses cannot be settled on time across provinces. These performances of the insured show that there are deficiencies in the way, content, and timing of BMIS information publicity in China (16). A survey of 970 respondents in Heilongjiang Province on their knowledge of BMIS policies showed that 77.1% had average or no knowledge of the policies (81), thus confirming the above view. Therefore, effective publicity methods should be actively explored to improve the insured's understanding of BMIS.

##### 4.2.3.2. Misinterpretation of information

Information misinterpretation occurs when the insured make a wrong interpretation of BMIS information. We found that the insured mainly focus on two aspects of misinterpretation: media information and the failure to understand the original intention for the exclusion of drugs from the basic medical insurance list.

This study shows that due to the limitations of knowledge and experience, the insured are easily misled by information publicized by the media. An interviewee of this study said that “the news propaganda indicates that BMIS can reimburse more than 70%, but the actual hospitalization compensation was only ~50%,” which made him very disappointed. The media selectively presents the best policy information or directly exaggerates the policy effect (82). Unfortunately, this strategy reduces people's evaluation of the policy, because such reports blindly raise people's expectations, and it should therefore be avoided.

The study also shows that some insured had low PBBMI owing to the sudden exclusion of their habitual drugs from the basic medical insurance list. We think that is highly correlated with a lack of understanding of the drug withdrawal mechanism. At the beginning of the 21st century, China's approval of drugs was relatively loose, resulting in the inclusion of many drugs with low cost performance and insufficient curative effects, and replaceable drugs in the list (83), which not only increased the expenditure pressure of basic medical insurance funds, but also affected the life and health of the insured. Following the reform of the healthcare system, China has gradually began adopting pharmacoeconomic evaluation methods to systematically evaluate and adjust drugs in the list and those intended to be included in the list (84). Accordingly, a total of 179 drugs have been transferred out of the list from 2019 to 2020 to make space for more good and new drugs (85, 86). However, the insured are not familiar with the original purpose of the list adjustment, but are mostly influenced by the increased economic pressure of purchasing drugs at their own expense and the distrust of the treatment effect of low-cost drugs, making them mistakenly believe that the core of the list adjustment is mainly to save basic medical insurance funds rather than for the personal interests of the majority of the insured, further resulting in their low PBBMI.

##### 4.2.3.3. Misjudgment of roles and functions

This study shows that insured relatively misjudged their roles and the functions of a basic medical insurance card.

Participation can increase people's emotional engagement and favorable feeling toward the event they are involved in Reynolds and Beresford (87). However, this study shows that some insured are relatively indifferent to participating in the supervision of basic medical insurance funds, which may be because they find it difficult to link the consequences of insurance fraud with their own interests. It is not a legal obligation to participate in supervision. However, this attitude indirectly reflects that they do not care enough about the BMIS. The lack of participation and emotional investment will relatively affect their PBBMI.

According to the interviewees' descriptions, we found that some insured's PBBMI was reduced because their basic medical insurance card could not buy non-medical supplies in the drugstore. As in most regions of China, there are certain historical reasons why these interviewees in Harbin hold this misunderstanding; China's regulatory system is not perfect and lacks supporting regulatory technical support (88). Many pharmacies long used the method of drug exchange to meet their own interests and the needs of the insured (89). This behavior amplified the insured's misperception of the payment function of the basic medical insurance card. Following China's crackdown on insurance fraud, the arbitrage of basic medical insurance cards by designated pharmacies has been effectively improved. However, the misperception among the insured is difficult to change in a short time.

#### 4.2.4. System environmental impact

PBBMI is a comprehensive perception formed by the integrated effects of various results. It includes not only the direct perceptions driven by the use of BMIS, but also the indirect perceptions generated by the system environment. In the interview, we found that when the insured evaluate PBBMI, it is easy to generate a halo effect (90). In other words, the insured will perceive the entire health system including BMIS according to their bad impression of other elements in the system environment.

##### 4.2.4.1. Poor performance of the health system

Our research shows that the important health system factors that indirectly affect PBBMI are doctors' poor work attitudes and defective hierarchical medical systems. Regarding doctors' attitudes, relevant studies indicate that Chinese doctors experience significant work pressure (91), lack of cultivation of humanistic quality and communication skills (92) and patients' distrust (35), which indeed drive some doctors' poor work attitudes (93, 94). Additionally, regarding the construction of hierarchical medical systems, owing to the imperfect differentiated reimbursement policy of BMIS and the inadequate capacity of primary care institutions (95, 96), many insured individuals in Harbin with common or frequently-occurring diseases still choose to go to large, overcrowded hospitals (97). These factors may have affected the insured's experience and negatively influenced their PBBMI.

##### 4.2.4.2. The elderly have difficulty adapting to the development of information technology in health care

Notably, the data survey in this study coincides with the outbreak of COVID-19. In this period, hospitals in all provinces and cities in China took many measures to manage the spread of COVID-19 and the gathering of people in hospitals, such as the widespread use of network appointment registration, online payment, and electronic report forms. However, these measures have inconvenienced the digital poor, represented in large part by the elderly. The elderly have considerable demand for medical services. However, China's Internet users aged 60 and over account for only 11.2% of the total relevant population; their information technology utilization rate is low (98). Moreover, the decline of physical function and the difficulty of accepting new things make it difficult for the elderly to use medical services in an information-based medical environment (99). The technological marginalization of the elderly results in poor experience of medical services. The effect of medical information technology on their health care utilization will shift and further influence their evaluation of BMIS and reduce the PBBMI.

## 5. Conclusions

The reform of China's BMIS is progressing vigorously. However, this study shows that 44% of the insured still have low PBBMI, and that the problem and obstacles for PBBMI mainly focus on the design of the BMIS, intuitive cognitive bias, rational cognitive bias, and system environmental influences.

Therefore, based on the quantitative and qualitative research results, the study indicates that to improve the insured's PBBMI, the government should focus on the insured who enroll for the URRBMI, receive low-income, lack recent basic medical insurance use experience and are elderly. Additionally, at the problem level, the obstacles should be eliminated from three aspects: improving the BMIS policy, reducing the cognitive bias of the insured and optimizing the health system environment. Regarding the policy of BMIS, the government should adjust drug prices, optimize the health technology evaluation, expand the scope of BMIS reimbursement, reduce the participation costs and advancing hospitalization costs for low-income people, reinforce the supervision of the basic medical insurance fund, and improve the drug supply guarantee mechanism to improve the service quality of BMIS. Regarding the intuitive and rational cognitive bias of the insured, the government should improve the publicity channels, contents, forms, and feedback channels of BMIS information to improve the cognitive of the insured. Moreover, the government can cooperate with the media platform to ascertain and clarify false information. Regarding, the health system environment, the government should adjust the workload of doctors, reinforce the cultivation of doctors' humanistic quality, and improve the construction of the hierarchical treatment system to improve the insured's medical experience. Additionally, these institutions that are related to BMIS should pay more attention to the actual difficulties of the elderly when providing services.

## 6. Limitations

This study had several limitations. First, owing to the effect of the COVID-19, the participation of rural migrants and respondents with recent basic medical insurance use experience was low. Second, due to resource and condition constraints, the quantitative survey sample can only represent the situation of Harbin, and cannot be extrapolated to the whole country. To reflect the situation in China as a whole, further studies involving a wider range of regions and populations are needed. Having acknowledged these limitations however, we are of the view that results and findings from this study provides a reference for other areas where the cause of medical insurance is undergoing reform.

## Data availability statement

The original contributions presented in the study are included in the article/Supplementary material, further inquiries can be directed to the corresponding authors.

## Author contributions

PW, LS, YL, and QW: conceptualization and manuscript revision. MJ, NN, and LG: data collection. PW, SL, and ZW: data analysis and original draft writing. PW, YZ, and WH: interpretation of data. All authors read and approved the final manuscript.
