# Supplementary material for: Perceptions of the benefits of the basic medical insurance system among the insured: a mixed methods research of a northern city in China
Source: Front Public Health. 2023 Apr 17;11:1043153. doi: 10.3389/fpubh.2023.1043153 (PMC10149763; doi:10.3389/fpubh.2023.1043153)
Supplement: Supplementary file 1 [file Table_1.DOC]

**Supplementary file 1**

**Supplementary Table 1 Qualitative Research Findings**

| **Tertiary theme** | **Secondary theme** | **Primary theme** | **Illustrative quotes from enrollee interviews** |
| --- | --- | --- | --- |
| Basic medical insurance system design | Inadequate coverage of services and guarantees | High drug price | *I think the price of these imported drugs such as German and American drugs, are not cheap. (Material 1)*  *I spend about 8,000 RMB yearly on oral medication. I feel that the price of medication is relatively high. (Material 15)*  *I have Parkinson’s disease; the relevant medication is very expensive; it costs 2000 monthly. However, my salary is not enough. This situation is very stressful. (Material 11)* |
|  |  | Inadequate reimbursement of drugs and medical consumables | *My mother has gallbladder cancer. The relevant medication is not reimbursed by basic medical insurance. Therefore, we are under great financial pressure. (Material 3)*  *Surgery requires various high-value medical consumables. However, they are not reimbursed by basic medical insurance. I hope that basic medical insurance will reimburse me.(Material 14)* |
|  | Insufficient attention to the needs of low-income people | The burden of participation costs | *I pay approximately 300 yuan yearly for premiums of urban and rural resident basic medical insurance, which is relatively high for us (farmers). Furthermore, I have a hard time accepting that the premiums go up yearly. (Material 9)*  *Urban employee basic medical insurance premiums are going up yearly. Moreover, we do not have more money to pay for it. (Material 18)* |
|  |  | The burden of advancing payment of hospitalization costs | *My family is really poor. My husband has to pay 30000 yuan in advance to make a heart stent. I can only borrow money from my sister. If the money is not paid in advance, the hospital will not treat him. (material 28)*  *I met a very sick person in the hospital who decided to give up his treatment because he did not have the money to pay for the hospital bill in advance. (material 20)* |
|  | Lack of regulatory measures and support | Lack of supervision and intervention in the risk of irregularities by medical institutions and doctors | *Nowadays, hospitals let you stay for half a month at most and then you have to go home, when it seems that the disease is well, but it is not cured at all when you go home, my mother experienced these things when she was hospitalized. (Material 23)* |
|  |  | Lack of guarantee of drug supply | *I cannot buy some general medicine at the hospital, which is the case in many hospitals. Moreover, I have to go to outside pharmacies to buy them. (Material 2)* |
| Intuitive cognitive bias | non-status quo reference point selection | Expect the cost of participation will remain the same and hospitalization will be fully reimbursed | *I think it would be good if the cost of participation did not increase. (Material 8)*  *I think the hospitalization expenses should be fully reimbursed by the basic medical insurance. (Material 9)* |
|  | Availability heuristics | Reliance on relatives and friends | *I get some information about basic medical insurance from my relatives and friends. (Material 1)* |
|  | Representativeness heuristics | Doctors’ omniscient Inference | *I am not familiar with the basic medical insurance system. I listen to the doctor’s advice, because they all know. (Material 2)* |
| Rational cognitive bias | Lack of information | Incomplete access to effective information about limited payment policy | *The basic medical insurance policy of “Zoledronic Acid Injection” stipulates that only people with fractures can be reimbursed. I cannot be reimbursed because I only have osteoporosis. I do not know this rule and I do not think this rule is very humanized. (material 3)* |
|  |  | Information update lag about medical treatment in non-residential places | *My father was hospitalized in Sanya in 2019. He needs to take all relevant materials back to the insured place before he can be reimbursed, which is very troublesome. (material 22)* |
|  | Misinterpretation of information | misleading by the media | *The news propaganda indicates that basic medical insurance can reimburse more than 70%, but the actual hospitalization accounts for only approximately 50%, because there are many drugs and services that are not reimbursed. The medical insurance policy is not as effective as advertised. (Material 14)* |
|  |  | The quality signals released by the basic medical insurance catalog adjustment are not effectively received | *“Alprostadil” was excluded from the basic medical insurance list. Moreover, doctors are now replacing it with a very low priced drug, whose effect I do not believe. Such a reform is aimed at saving funds. (Material 10)* |
|  | Misjudgment of role and function | Participating in Indifference | *I feel that other people’s fraudulent insurance practices have affect me insignificantly. (Material 22)*  *Why should I supervise those things (fraudulent insurance)? It has nothing to do with me. (Material 23)* |
|  |  | Basic medical insurance card function misjudgment | *If the basic medical insurance card had more money, I would buy some daily necessities, such as soybean oil. (Material 26)* |
| System environmental | Health system performance | Poor attitude of doctors | *During my visit to a certain hospital, the doctor did not take the initiative to explain anything related to basic medical insurance to me. I therefore inquired. Unfortunately, he was very impatient and did not explain clearly. (Material 26)* |
|  |  | The hierarchical medical system is ineffective | *Generally, I would not go to primary medical institutions even if the reimbursement rate is high. I would still prefer a larger hospital because it has better equipment and doctors. (Material 22)* |
|  | Medical Information Development | Older adults have difficulty adapting to health care information technology | *Most of the hospitals have implemented information technology, such as cell phone registration. However, we do not know how to operate these. (Material 12)*  *Nowadays, information technology is so common in hospitals that it is too difficult for elderly people to go to the hospital for treatment. (Material 10)* |
